# Dietary Patterns and Socioeconomic Status in the Very Old: The Newcastle 85+ Study

**DOI:** 10.1371/journal.pone.0139713

**Published:** 2015-10-21

**Authors:** Antoneta Granic, Karen Davies, Ashley Adamson, Thomas Kirkwood, Tom R. Hill, Mario Siervo, John C. Mathers, Carol Jagger

**Affiliations:** 1 Newcastle University Institute for Ageing, Newcastle upon Tyne, United Kingdom; 2 Institute of Health & Society, Newcastle University, Newcastle upon Tyne, United Kingdom; 3 Human Nutrition Research Centre, Newcastle University, Newcastle upon Tyne, United Kingdom; 4 Institute for Cell and Molecular Biosciences, Newcastle University, Newcastle upon Tyne, United Kingdom; 5 School of Agriculture, Food and Rural Development, Newcastle University, Newcastle upon Tyne, United Kingdom; 6 Institute of Cellular Medicine, Newcastle University, Newcastle upon Tyne, United Kingdom; University of Valencia, SPAIN

## Abstract

**Background:**

Dietary patterns (DP) are associated with health outcomes in younger adults but there is a lack of evidence in the very old (aged 85+) on DP and their association with sociodemographic factors, lifestyle, health and functioning measures. Higher socioeconomic status (SES) has been linked with healthier DP but it is not known whether these associations are sustained in the very old.

**Objective:**

We aimed to (a) characterise DP in the very old and (b) assess the relationships between three SES indicators (education, occupational class and area-deprivation index [IMD]) and DP.

**Methods:**

Complete dietary data at baseline (2006/07) for 793 participants in the Newcastle 85+ Study were established through 24-hr multiple pass recall. We used Two-Step clustering and 30 food groups to derive DP, and multinomial logistic regression models to assess the association with SES.

**Results:**

We identified three distinct DP (characterised as ‘High Red Meat’, ‘Low Meat’, and ‘High Butter’) that varied with key sociodemographic, health and functioning measures. ‘Low Meat’ participants were more advantaged (i.e. higher education and occupational class, and lived in more affluent areas in owned homes), were least disabled, cognitively impaired, and depressed, and were more physically active than those in the other DP. After adjusting for other lifestyle factors, cognitive status and BMI, lower educational attainment remained a significant predictor of ‘High Red Meat’ and ‘High Butter’ membership compared with ‘Low Meat’ (‘High Red Meat’: OR [95% CI] for 0–9 and 10–11 years of education vs. ≥12 years: 5.28 [2.85–9.79], p<0.001 and 3.27 [1.65–6.51], p = 0.001, respectively; ‘High Butter’: 3.32 [1.89–5.82], p<0.001 and 2.83 [1.52–5.28], p = 0.001).

**Conclusions:**

In this cohort of very old adults, we detected a favourable DP (‘Low Meat’), which was associated with better health and functioning and higher SES.

## Introduction

The importance of whole diet, dietary patterns (DP) and the consequent synergistic effects of food combinations (rather than a single food or nutrient) on healthy ageing, disease prevalence and survival in older adults is increasingly recognised [1–9]. Greater adherence to healthy DP in later life established by either dietary scores (e.g. Mediterranean-style diet [MeDi] and Dietary Approaches to Stop Hypertension [DASH]) or derived from primary data through dimension-reduction techniques have been linked with decreased risk of cardiovascular diseases [1–3], cancer [1], stroke [4], dementia [5,6], and all-cause and disease-specific mortality [1,7–9]. Healthier DP are generally characterised by higher than average intake of beneficial foods (e.g. fruits and vegetables, fish, low-fat dairy, and whole grains), and lower intake of potentially less healthy foods (e.g. red and processed meats, refined cereals, and confectionery and desserts), which translates into a more nutrient-dense diet with lower energy density, and more favourable nutrient status [9–12].

Several individual, social and environmental factors including age, gender, ethnicity, self-perceived health, diseases burden, social support, and socioeconomic status (SES) are determinants of diet quality in both the general, and older, adult populations [13–25]. In particular, diet quality is associated with SES operationalised as education, occupation or income [10,13,14,18,21–25]. For example, a higher educational attainment has been positively associated with healthier ‘vegetable-based’ DP and lower levels of education with less healthy ‘sweet- and fat-dominated’ DP among older adults (aged 60+) from nine European countries participating in the European Prospective Investigation into Cancer and Nutrition (EPIC)-Elderly study [10]. In the Québec NuAge study, older adults (aged 67 to 84) with higher educational attainment and better diet knowledge had higher diet quality assessed by the Canadian Healthy Eating Index [14]. Similarly, in the 2003–2004 National Health and Nutrition Examination Survey older adults (aged 65+) with the highest education (especially college graduates) had higher dietary scores based on the 2005 Dietary Guidelines for Americans, and higher intake of fruits, vegetables, whole grains and alcohol [18]. However, diet quality and adherence to healthy DP in relation to SES among the very old (aged 85+) has not been explored. In addition, very little is known about associations between DP and health and functioning status, and social, socioeconomic, and behavioural factors in this age group.

The main advantage of DP analysis in investigating the relationship between diet and healthy ageing is the ability to capture the complexity of human diet and to account for synergistic, additive and cumulative effect of different foods and nutrients on health. The most common approach used to derive DP is an *a priori*, hypothesis-driven approach which assesses the level of adherence to a pre-defined healthy diet score (index) for a specific diet (i.e. MeDi and DASH) or dietary guidelines for general health (i.e. Healthy Eating Index, Healthy Diet Indicator) [26–28]. Limitations of an *a priori* approach include (a) the reliance on current scientific evidence on what constitutes a healthy diet or on dietary guidelines which may not be accurate for the very old, and (b) the inability of dietary scores to capture the effect of the whole diet on health. On the other hand, *a posteriori* DP are purely data-driven and do not rely on the current state of knowledge in nutrition. They are derived by statistical methods such as factor or cluster analysis but, by being based on the total diet, may provide better characterisation of the habitual diet of a specific population group. Specifically, cluster analysis creates a latent variable by grouping individuals into distinct, non-overlapping clusters based on homogeneity of foods and nutrients consumed [29].

Despite extensive interest among the scientific community and the general public about the potential benefits of diet for heath and functioning in older adults (including the very old), there is limited epidemiological evidence about dietary habits and the determinants thereof amongst the very old. Furthermore, most studies investigating the role of diet/DP in healthy ageing in later life used an *a priori* approach and only a few derived DP empirically or included the very old despite this age group now being the most rapidly increasing sector of the population [e.g. 10,14,20,23].

To the best of our knowledge, the present study is the first cohort study to (a) describe the habitual diet of very old adults using an *a posteriori* approach (cluster analysis); and (b) determine how DP vary by sociodemographic, lifestyle, health and functioning measures.

## Methods

### Subjects

Participants were drawn from the Newcastle 85+ Study, a prospective study of over 1,000 individuals belonging to a single-year birth cohort (1921), who were recruited through general practices in Newcastle and North Tyneside, UK [30,31]. In brief, the study aimed to determine the health trajectories and bio-psychosocial factors (including diet) that contribute to the maintenance of physical and mental function and independence of the very old (aged 85+). Research nurses completed a health assessment (i.e. questionnaires, measurements, function tests and collection of fasting blood samples) in each participant’s usual residence, including institutions. General practice (GP) medical records were reviewed for diagnosed diseases and prescribed medication. At baseline (2006/07), both health assessment and GP records data were obtained for 845 participants (58.2% of those eligible to participate), and a further 188 had GP record review only. In all, 805 participants consented to dietary assessment, and 793 (98.5%) completed two 24-hour multiple-pass dietary recall (24-hr MPR) [32] conducted on two non-consecutive days, and were used to derive DP. The analytic sample comprised of 791 participants who had complete diet, health assessment and GP records data at baseline.

### Ethics statement

The study was approved by the Newcastle & North Tyneside Local Research Ethics Committee 1. Written informed consent was obtained from each participant or from a consultee (usually a relative or carer) if an individual lacked capacity to consent.

### Measurements

#### Assessment of dietary intake

The methodology of dietary assessment in this cohort has been described in detail elsewhere [33]. Briefly, habitual diet of the Newcastle 85+ participants was evaluated at baseline by trained research nurses using the 24-hr MPR. Prior comparison of two different methods of dietary assessment in a sub-sample (n = 89) of this cohort determined that: (a) 24-hr MPR provides more accurate estimates of energy and nutrient intakes compared with a food frequency questionnaire (FFQ), and (b) 24-hr MPR is an acceptable dietary assessment approach for the very old [33].

The 24-hr MPR method has been extensively used in national surveys in the US and the UK [32,34], and it is suitable for individual dietary assessment minimizing participant burden. Compared with weighted food records or food diaries, 24-hr MPR (paper-pencil and automated version) has been accepted as a more accurate measure of diet and individual energy intake [32–34]. The method captures individuals’ diet by recording detailed food intake during the previous day (i.e. food amount, type and brand, cooking method, meal occasion/time).

Dietary intake was assessed on two different days of the week at least one week apart, with some exceptions (i.e. 23% of participants were interviewed on the same day of the week, and 25.5% had interviews <7 days apart). There were no dietary recalls for Fridays and Saturdays. Portion sizes were estimated from the description on the food packet, or aided by use of a photographic atlas, which includes images of household measuring tools [35]. The data on foods consumed were coded based on McCance and Widowson’s food composition tables [36]. Over 2,000 unique food codes were used and entered into a Microsoft Access-based dietary database. Individual foods were then grouped into 118 distinct food groups that had been previously established by the Human Nutrition Research Centre at Newcastle University. Each food group represented an average weight (in grams) of foods consumed on both days of measurement. The food groups were further collapsed into 33 broader groups based on food and nutrient composition similarities, and then categorised as absent or present in food intake (coded 0 and 1, respectively). Of these 33 food groups, 30 were used in the cluster analysis (S1 Table) and contributed the most to DP separation. The Access database also provided information on food weight, and estimates of nutrient and energy intake for all 118 food groups.

#### SES measures

SES was determined using three independent measures over the lifespan, which were coded into three levels (low [0], medium [1] and high [2]): (a) for early life: years of full-time education attained (9 years or less / 10–11 years / 12 years or more for low, medium and high respectively); (b) for mid to late adulthood: previous main occupation coded to the National Statistics Socio-economic Classification (NS-SEC) system [37] combined into three classes (routine and manual occupation / intermediate occupations / higher managerial, administrative and professional occupations), and (c) for very late adulthood: level of poverty in current area of residency defined by the Index of Multiple Deprivation (IMD) [38] (referred to later in the text as ‘deprivation index’), and categorised into quartiles and reverse-coded (>75^th^ percentile [Q4: ‘poor areas’] / 25^th^-75^th^ percentile [Q2+Q3: ‘intermediate group’] / <25^th^ percentile [Q1: ‘affluent areas’]).

Previous main occupation (defined as a job or role a participant performed for longest time in his/her working life) was established from the job history of each participant, except for 265 women (33.5% of analytic sample) who had their occupational class taken from husbands’ occupation. The IMD is a composite, weighted measure of multiple deprivations in seven domains (income, employment, health and disability, education, housing and services, living environment, and crime) for small geographic areas of about 1,500 inhabitants [38].

#### Other measures and covariates

The characteristics of participants in each of the DP were compared using data collected concurrently with diet: (a) sociodemographic (sex; marital status [not married / married]; type of home [rented / owned/mortgaged / live in institutions]); (b) lifestyle (diet change in past year [yes / no]; smoking [never / current smoker / former smoker]; physical activity [low/moderate/high]; current alcohol intake [yes / no], and (c) health-related factors (number of chronic diseases [0–1 / 2 / ≥3], including prevalence of individual diseases, and *APOE ɛ4* status [no *ɛ4* allele / 1+ *ε4* alleles]; body mass index (BMI) [underweight (<18.5) / normal (>18.5–25) / overweight (>25–30) / obese (>30)]), cognitive status [normal (≥26 points on Mini Mental State Examination, MMSE) / impaired (≤25 MMSE)] [39], and supplement intake [yes / no]).

#### Statistical analysis

**Cluster analysis:** DP were established using participants with two complete 24-hr MPR at baseline (n = 793). Two participants had incomplete health/GP records data and were excluded from multivariate analyses. The non-responders (i.e. those refusing or with dietary protocol violation; n = 54) were compared with the 791 participants (analytic sample) using independent t test for continuous and χ^2^ test for categorical variables.

DP were derived using the SPSS Two-Step cluster analysis [40–43]. The procedure is suitable for large data sets and accommodates both categorical and continuous variables. It uses one pass through the data to create small pre-clusters based on a log-likelihood distance criterion (Step 1), which are merged into distinct dietary groups using agglomerative hierarchical clustering method (Step 2). We used automatic selection and the Bayesian Information Criterion (BIC) to determine the optimal number of DP and the best DP solution, which was achieved with 30 food groups (Table 1). Other criteria included: (a) number of food groups with high importance factor (IF) that most readily separated the DP, excluding those which had IF <0.01 consistently; (b) ratio size between the DP; and (c) interpretability of the DP solution. The robustness and stability of the final DP solution was re-evaluated by random ordering of cases (four times) and by comparing DP solution characteristics.

**Table 1:** Percentages of participants consuming each of 30 food groups by dietary pattern (DP) with importance factor*.

Food group†	DP1: High Red Meat	DP2: Low Meat	DP3: High Butter	Importance factor (IF)
	*n* = 277	*n* = 260	*n* = 256	range: 0–1
Fruits	71.1%	88.8%	77.3%	0.06
Vegetable	90.6%	72.7%	89.5%	0.09
**Potato and potato dishes**	95.3%	58.1%	90.6%	**0.31**
**Legumes**	54.2%	15.0%	49.6%	**0.22**
Nuts	2.2%	11.2%	8.6%	0.04
Refined grains and cereal products	84.5%	74.6%	85.9%	0.03
Whole grains and cereal products	84.8%	89.6%	76.6%	0.04
Fish and sea food	27.4%	45.8%	29.7%	0.05
**Red meats and meat dishes**	88.4%	44.6%	68.8%	**0.26**
Bacon and ham	46.2%	44.2%	58.6%	0.03
Poultry	41.2%	23.8%	35.9%	0.04
Processed and other meats	22.0%	14.6%	27.0%	0.03
Eggs	32.9%	45.4%	38.7%	0.02
Soups	19.5%	33.1%	18.0%	0.04
**Butter**	7.9%	33.8%	98.4%	**1.00**
Saturated fat spreads and margarine	24.9%	17.3%	7.8%	0.06
**Unsaturated fat spreads and oils**	70.0%	60.8%	5.5%	**0.56**
**Gravy**	54.6%	5.8%	44.1%	**0.34**
Low fat dairy	58.8%	70.8%	43.0%	0.09
High fat dairy	53.1%	77.3%	60.5%	0.08
Preserves and syrups	45.5%	62.7%	51.2%	0.04
Chocolate	28.5%	39.6%	30.1%	0.02
Biscuits and cakes	84.1%	83.5%	86.3%	<0.02
Deserts and sweets	68.6%	63.1%	62.9%	<0.02
**Snacks and savouries**	17.3%	46.2%	29.3%	**0.11**
Tea	94.9%	91.2%	93.0%	<0.02
**Coffee**	40.4%	70.4%	40.6%	**0.14**
Alcohol	23.5%	44.2%	35.2%	0.06
Hot drinks	12.3%	16.8%	11.7%	<0.02
Soft sugary drinks	41.2%	31.5%	39.1%	<0.02

*DP were derived using the SPSS Two-Step clustering of 30 food groups (coded as binary variable: absence or presence of food group). Seven hundred and ninety-three participants had complete dietary data and were used in cluster analysis.

^†^Eight food groups contributing the most to dietary pattern separation are indicated in bold.

**Nutritional characteristics by dietary patterns:** Intakes in grams/day of 10 food groups with the highest IF across DP were compared using the Kruskal-Wallis test (S2 Table). Intakes of selected nutrients and of energy (including percent energy from nutrients) (Table 2) by DP were compared by ANOVA (with post hoc Tukey HDS or Games-Howell) for normally distributed and Kruskal-Wallis for non-normally distributed variables. The nutrient contents of all foods consumed by participants belonging to each DP were included in these analyses.

**Table 2:** Intakes of selected nutrients and energy consumption per day by dietary patterns.

Outcome (M, SD)*	DP1: High Red Meat	DP2: Low Meat	DP3: High Butter	p^†^
Food weight (g)	2387 (640)	2333 (628)	2297 (612)	
Total energy (KJ)	7077 (2214)	6933 (2136)	7126 (2025)	
Total Energy (Kcal)	1685 (528)	1652 (510)	1699 (483)	
Sex-specific quartiles of total energy % (n)^‡^				
Q1	28.3 (78)	25.4 (66)	20.8 (53)	
Q2	23.6 (65)	24.2 (63)	27.5 (70)	
Q3	23.6 (65)	27.3 (71)	24.7 (63)	
Q4	24.6 (68)	23.1 (60)	27.1 (69)	
Food energy (KJ)	6937 (2168)	6699 (2036)	6961 (1955)	
Food energy (Kcal)	1657 (518)	1600 (486)	1663 (467)	
Food energy density (KJ/g)	3.0 (0.8)	2.9 (0.8)	3.1 (0.7)	<0.001
Fat (g)	66.5 (26.3)	67.7 (26.8)	73.7 (26.4)	0.004
% Energy from fat	35.0 (6.4)	36.9 (7.0)	38.9 (6.8)	<0.001
Protein (g)	68.1 (23.5)	60.7 (21.0)	62.7 (21.4)	<0.001
% Energy from protein	17.0 (3.9)	15.6 (3.3)	15.4 (3.4)	<0.001
Carbohydrates (g)	207.2 (68.9)	197.3 (62.3)	197.2 (59.8)	
%Energy from carbohydrates	47.9 (6.2)	47.5 (7.0)	45.5 (6.8)	<0.001
Starch (g)	111.1 (39.0)	100.0 (38.8)	104.9 (33.0)	
%Energy from starch	25.9 (5.4)	24.0 (5.7)	24.4 (5.3)	<0.001
NMES (g)	48.9 (32.8)	48.3 (27.5)	47.9 (33.0)	
%Energy NMES	10.9 (5.8)	11.5 (5.6)	10.7 (6.4)	
Total sugars (g)	92.3 (41.2)	94.6 (38.0)	89.4 (41.4)	
%Energy from total sugars	21.1 (6.3)	22.8 (6.9)	20.4 (7.1)	<0.001
NSP (Englyst method)^§^ (g)	11.3 (5.4)	11.0 (5.2)	10.4 (4.7)	
Cholesterol (mg)	181.0 (112.1)	190.6 (126.8)	230.5 (124.0)	<0.001
MUFA (g)	15.6 (7.8)	16.7 (8.2)	18.1 (8.0)	<0.001
PUFA (g)	8.0 (5.2)	8.0 (5.3)	6.2 (4.1)	<0.001
SFA (g)	22.7 (11.0)	25.1 (12.8)	31.5 (12.7)	<0.001
MUFA/SFA ratio	0.7 (0.2)	0.7 (0.3)	0.6 (0.2)	<0.001
Alcohol (g)	4.8 (12.2)	8.1 (14.9)	5.7 (12.0)	<0.001
Water (g)	1981.4 (567.2)	1932.4 (556.9)	1895.1 (539.8)	

KJ, kilojoules; Kcal, kilocalories; NMES, non-milk extrinsic sugars; NSP, non-starch polysaccharides; MUFA, monounsaturated fatty acids; PUFA, polyunsaturated fatty acids; SFA, saturated fatty acids.

*Normality of data was tested using skewness and kurtosis statistics, histograms and Q-Q plots. Values are means (M, SD) or percentages (%), which were estimated based on consumption of all 118 food groups [36].

^†^ANOVA (with Post Hoc Tukey HDS or Games-Howell) was used for normally distributed and Kruskal-Wallis for non-normally distributed continuous variables. Only significant *p* values are reported.

^‡^The range for total energy quartiles (Kcal) are: Q1 (559–1581), Q2 (1588–1909), Q3 (1909–2283), and Q4 (≥2287) for men, and Q1 (513–1219), Q2 (1221–1490), Q3 (1493–1756), and Q4 (≥1761) for women.

^§^Englyst method measures NSP and is the most commonly used method in the UK.

**Blood lipid profile by dietary patterns:** Blood lipids concentrations (i.e. total cholesterol, LDL-cholesterol, and HDL-cholesterol in mmol/L) of participants in each DP group are summarised in Table 3. Blood collection was carried out concurrently with dietary assessment as described previously [44].

**Table 3:** Blood lipid profile of study participants by dietary patterns.

Outcome (M, SD)*	DP1: High Red Meat	DP2: Low Meat	DP3: High Butter	p^†^
Total cholesterol (mmol/L)	4.7 (1.2)	4.5 (1.3)	4.9 (1.2)	0.04
HDL-cholesterol (mmol/L)	1.5 (0.4)	1.6 (0.4)	1.5 (0.4)	<0.001
LDL-cholesterol (mmol/L)	2.6 (1.1)	2.8 (1.1)	2.8 (1.0)	
Total cholesterol/HDL ratio	3.4 (1.0)	3.31 (1.0)	3.4 (1.0	

HDL, high density lipoprotein; LDL, low density lipoprotein.

* Normality of data was tested using skewness and kurtosis statistics, histograms and Q-Q plots.

^†^ANOVA (with Post Hoc Tukey HDS or Games-Howell) was used for normally distributed and Kruskal-Wallis for non-normally distributed continuous variables. Only significant p values are reported.

**Sociodemographic and health characteristics by dietary patterns:** The sociodemographic and health characteristics of the participants in each DP group were compared using the Kruskal-Wallis test for ordered and χ^2^ test for categorical variables (Table 4 and S3 Table). All statistics were 2-sided at α = 0.05.

**Table 4:** Baseline characteristics of study participants by dietary patterns.

Characteristic	All participants*	DP1: High Red Meat	DP2: Low Meat	DP3: High Butter	p^†^
	n = 791	n = 276	n = 260	n = 255	
**Socio-demographic factors**					
Women % (n)	61.8 (489)	57.6 (159)	64.6 (168)	63.5 (162)	
Marital status % (n)					0.02
Not Married	69.4 (548)	66.9 (184)	76.0 (233)	65.5 (167)	
Married	30.5 (241)	33.1 (91)	23.9 (62)	34.5 (88)	
Years of education					<0.001
0–9	64.1 (501)	74.6 (203)	51.9 (134)	65.3 (164)	
10–11	23.4 (183)	19.1 (52)	25.2 (65)	26.3 (66)	
≥12	12.4 (97)	6.3 (17)	22.9 (59)	8.4 (21)	
Occupational class % (n)					<0.001
Routine/manual professions	51.1 (305)	58.1 (151)	39.4 (100)	56.1 (134)	<0.001
Intermediate professions	14.5 (109)	14.2 (37)	15.0 (38)	14.2 (34)	
Higher managerial/administrative	34.4 (259)	27.7 (72)	45.7 (116)	29.7 (71)	
Index of multiple deprivation % (n)					0.003
Poor areas	24.3 (192)	28.6 (79)	16.5 (43)	27.5 (70)	
Intermediate	50.2 (397)	48.9 (135)	51.5 (134)	50.2 (128)	
Affluent areas	25.5 (202)	22.5 (62)	31.9 (83)	22.4 (57)	
Type of home % (n)					<0.001
Rented	34.1 (269)	35.9 (99)	29.5 (76)	36.9 (94)	
Owned/mortgaged	57.0 (450)	50.7 (140)	68.1 (177)	52.2 (133)	
Live in institutions	8.9 (70)	13.4 (37)	1.9 (5)	11.0 (28)	
**Lifestyle factors**					
Diet change in past year % (n)					
Yes	6.9 (53)	5.7 (15)	8.1 (21)	6.7 (17)	
Supplement intake % (n)					0.04
Yes	42.6 (337)	38.8 (107)	48.8 (127)	40.4 (103)	
Smoking % (n)					0.04
Never	35.3 (279)	40.2 (111)	33.1 (86)	32.3 (82)	
Current smoker	5.7 (45)	3.6 (10)	5.0 (13)	8.7 (22)	
Former smoker	59.0 (466)	56.2 (155)	61.9 (161)	59.1 (150)	
Physical activity % (n)					0.002
Low	22.3 (176)	27.3 (75)	14.2 (37)	25.2 (64)	
Moderate	43.5 (343)	42.5 (117)	46.5 (121)	41.3 (105)	
High	34.2 (270)	30.2 (83)	39.2 (102)	33.5 (85)	
Current alcohol intake % (n)					0.002
Yes	59.9 (474)	54.3 (150)	68.5 (178)	57.3 (146)	
**Health-related factors**					
Number of chronic diseases % (n)					0.001
0–1	29.5 (233)	24.6 (68)	30.0 (78)	34.1 (87)	
2	30.1 (238)	34.1 (94)	26.9 (70)	29.0 (74)	
3 and more	40.5 (320)	41.3 (114)	43.1 (112)	36.9 (94)	
Number of disabilities % (n)					0.001
Not known disabilities	20.4 (161)	17.0 (47)	24.2 (63)	20.0 (51)	
1–6	52.1 (412)	50.0 (138)	56.9 (148)	49.4 (126)	
7–12	18.7 (148)	21.4 (59)	13.8 (36)	20.8 (53)	
13–17	8.8 (70)	11.6 (32)	5.0 (13)	9.8 (25)	
BMI^‡^ % (n)					0.02
Underweight (<18.5)	6.1 (48)	4.3 (12)	6.2 (16)	7.8 (20)	
Normal (>18.5–25)	55.0 (435)	51.4 (142)	57.3 (149)	56.5 (144)	
Overweight (>25–30)	29.8 (236)	31.2 (86)	30.4 (79)	27.8 (71)	
Obese (>30)	9.1 (72)	13.0 (36)	6.2 (16)	7.8 (20)	
Depressive symptoms^§^ % (n)					0.009
0-5/none	75.3 (591)	72.7 (197)	81.5 (212)	71.7 (182)	
6-7/mild	12.5 (98)	13.3 (36)	10.4 (27)	13.8 (35)	
≥8/severe	7.8 (61)	7.7 (21)	6.9 (18)	8.7 (22)	
MMSE <15	4.5 (35)	6.3 (17)	1.2 (3)	5.9 (15)	
Cognitive status % (n)					<0.001
impaired (≤25 MMSE score)	27.2 (214)	34.4 (94)	18.1 (47)	28.6 (73)	
normal (26–30)	72.8 (574)	65.6 (179)	81.9 (213)	71.4 (182)	

BMI, body mass index; MMSE, Mini-Mental State Examination; COPD, chronic obstructive pulmonary disease.

*Two participants with assigned dietary pattern did not have complete health assessments and GP records data, and were excluded from the analyses.

^†^Kruskal-Wallis test for ordered and non-normally distributed continuous variables and χ^2^ test for categorical variables. In the *post hoc* χ^2^ test analyses, adjusted residuals were used to determine which cells were major contributors to rejecting the null hypothesis at α = 0.05.

^‡^BMI was imputed with sex-specific means for 62 participants.

^§^Fifteen point Geriatric Depression Scale (GDS).

**SES determinants of DP membership:** We utilised multinomial logistic regression with stepwise forward entry to examine associations between DP and each SES indicator separately (Model 1), and together (Model 2), and adjusted for other lifestyle (physical activity and smoking) and health-related factors (BMI and cognitive status at baseline) (Model 3) (Table 5). In sensitivity analyses, we controlled additionally for type of home, and total energy (instead of BMI), and repeated all analyses in a sub-sample which excluded participants who lived in institutions (n = 70, 8.9%) (S4 Table).

**Table 5:** Socioeconomic determinants of dietary pattern membership*.

Dietary Pattern	SES indicator	Model 1^†^	p	Model 2^‡^	p	Model 3^§^	p
DP1: High Red Meat	Education (years)	OR (95% CI)		OR (95% CI)		OR (95% CI)	
	0–9	5.26 (2.94–9.41)	<0.001	5.55 (3.02–10.21)	<0.001	5.28 (2.85–9.79)	<0.001
	10–11	2.78 (1.45–5.33)	0.002	3.30 (1.67–6.49)	0.001	3.27 (1.65–6.51)	0.001
	≥12 (ref)	1		1			
	Occupational class						
	Routine/manual	2.43 (1.65–3.59)	<0.001				
	Intermediate	1.57 (0.91–2.69)	0.1				
	Managerial/administrative (ref)	1					
	Deprivation Index						
	Poor areas	2.46 (1.50–4.04)	<0.001				
	Intermediate	1.35 (0.90–2.03)	0.15				
	Affluent areas (ref)	1					
DP2: Low Meat (ref)		1		1		1	
DP3: High Butter	Education (years)						
	0–9	3.44 (1.99–5.95)	<0.001	3.42 (1.96–5.98)	<0.001	3.32 (1.89–5.82)	<0.001
	10–11	2.85 (1.56–5.22)	0.001	2.90 (1.56–5.39)	0.001	2.83 (1.52–5.28)	0.001
	≥12 (ref)	1					
	Occupational class						
	Routine/manual	2.19 (1.48–3.24)	<0.001				
	Intermediate	1.46 (0.84–2.53)	0.18				
	Managerial/administrative (ref)	1					
	Deprivation Index						
	Poor areas	2.37 (1.43–3.94)	0.001				
	Intermediate	1.39 (0.92–2.11)	0.12				
	Affluent areas (ref)	1					

ref, referent

*Multinomial logistic regression with stepwise forward entry

^†^each SES indicator entered separately

^‡^SES indicators entered together

^§^additionally adjusted for lifestyle (physical activity, smoking) and health-related factors (BMI, cognitive status)

We used the Kolmogorov-Smirnov test and Q-Q plots to determine normality of continuous variables, and multiple linear regression to evaluate covariates for multicollinearity and by inspecting correlation matrix, VIF tolerance and the condition index. All analyses were conducted using IBM SPSS (V.19 or 21; IBM Corporation, Armonk, NY, USA).

## Results

Comparison of the 54 participants without dietary data and the 791 participants with assigned DP and complete health assessments and GP records review revealed that those with missing data were more likely to live in institutions (p<0.001), to be cognitively impaired (p<0.001) or diagnosed with dementia (p<0.001), and to be more disabled (p<0.001) and less physically active (p = 0.002) (data not shown).

### Dietary patterns classification and differentiation

Using Two-Step clustering, a three-cluster solution proved to be optimal (Table 1) yielding approximately equal numbers of participants in each cluster. Eight food groups contributed the most to DP separation, notably butter, unsaturated fats spreads and oils, gravy, potato and potato dishes, red meats and meat dishes, and legumes. DP1 (n = 277), a ‘High Red Meat’ dietary pattern, was over-represented by red meats/meat dishes (88.4% of participants in this DP consumed red meats/meat dishes), gravy (54.6%), potato/potato dishes (95.3%), legumes (including baked beans) (54.2%) and unsaturated fats spreads (70.0%), and under-represented by butter (only 7.9% of them consumed butter). DP2 (n = 260), a ‘Low Meat’ dietary pattern, was under-represented by meat/meat dishes (44.6%), gravy (5.8%), potato/potato dishes (58.1%), and moderately represented by butter (33.8%). Compared with the others, this DP contained the highest percentage of participants reporting eating fruits, nuts, whole grains and cereal products, fish and sea food, eggs, soups, low and high fat dairy, coffee and alcohol. As a consequence, DP2 was regarded as the healthiest and was used as a referent in subsequent analyses. DP3 (n = 256), a ‘High Butter’ dietary pattern, was over-represented by butter (98.4%), moderately represented by red meats (68.8%), and under-represented in intake of unsaturated (5.5%) and saturated fats spreads (7.8%).

The robustness of these DP was corroborated when we compared percentage of intake (frequency) with g/day of intake of 10 food groups with the highest IF across DP (S2 Table). DP2 was characterised by higher intake (g/day) of healthy foods, and DP1 and DP3 by higher consumption of potentially less healthy foods.

### Nutritional characteristics by dietary patterns

The three DP did not differ according to total weight of food consumed or to intakes of total energy, food energy, carbohydrates, starch, total sugars, non-milk extrinsic sugars (NMES), non-starch polysaccharides (NSP) and water. Compared with DP1 and DP2, participants belonging to DP3 (‘High Butter’) had the highest consumption of total fat (p = 0.004), cholesterol, monounsaturated fatty acids (MUFA), and saturated fatty acids (SFA) (p<0.001), the lowest intake of polyunsaturated fatty acids (PUFA) (p<0.001) and MUFA/SFA ratio (p<0.001), the highest percent energy from fat (p<0.001) and SFA (p<0.001), and the highest food energy density (p<0.001), but the lowest percent energy from carbohydrates (p<0.001) (Table 2). Those in DP2 (‘Low Meat’) had the lowest intake of protein (p<0.001) and the highest consumption of alcohol (p<0.001), whereas DP1 (‘High Red Meat’) was highest in percent energy from protein (p<0.001) and starch (p<0.001).

### Blood lipid profile by dietary patterns

Compared with participants in DP1 and DP3, those in DP2 (‘Low Meat’) had the lowest total plasma cholesterol (p = 0.04) and the highest HDL-cholesterol concentration (p<0.001).

### Sociodemographic and health characteristics by dietary pattern

Compared with other DP, those in DP2 (‘Low Meat’) were less likely to be married (p = 0.02), were more educated (p<0.001), had higher managerial/administrative occupations (p<0.001), were more likely to own their homes (p<0.001), live in affluent areas (p = 0.003), be more physically active (p = 0.002), drink alcohol (p = 0.002), and were the least likely to be disabled (p = 0.001), to be obese (p = 0.02), to have dementia (p<0.001) or cognitive impairment (≤25 MMSE) (p<0.001), renal disease (p = 0.02), depressive symptoms (p = 0.009) or to be *APOE ɛ4* positive (p = 0.002) (Table 4 and S3 Table). Those in DP1 (‘High Red Meat’) were more likely to have never smoked (p = 0.04), but to be diagnosed with cardiovascular disease (p = 0.04) at baseline compared with the other DP.

### SES determinants of DP membership

Utilizing multinomial logistic regression, we examined the likelihood of belonging to a less healthy DP (‘High Red Meat’ [DP1] and ‘High Butter’ [DP3]) compared with the healthier DP (‘Low Meat’ [DP2]) based on SES indicators over the lifespan (i.e. education, occupation, and deprivation index) (Table 5). All three SES measures separately predicted DP1 and DP3 membership (Model 1). For example, participants with low education level (0–9 years) were more likely to belong to DP1 (OR [95% CI]: 5.26 [2.94–9.41], p<0.001) and DP3 (3.44 [1.99–5.95], p<0.001) compared with those with higher education attainment (≥12 years). Similar associations were observed for those living in poorer compared with more affluent areas (DP1: 2.46 [1.50–4.04], p<0.001; DP3: 2.37 [1.43–3.94], p = 0.001). However, in a model with all three SES indicators, only lower educational attainment (both low and middle) predicted ‘High Red Meat’ and ‘High Butter’ membership. This effect remained significant after adjusting for lifestyle (physical activity and smoking) and health-related factors (BMI and cognitive status at baseline) (e.g. OR [95% CI] for low vs. higher education for DP1: 5.55 [3.02–10.21], p<0.001; DP3: 3.42 [1.96–5.98], p<0.001) (Model 3).

Adjusting for total energy intake in the final model or additionally adjusting for type of home, sex and marital status did not change the associations between education and both less healthy DP (details not shown). Similar results were obtained when models were fitted to a sub-sample of participants after excluding those living in institutions (n = 70). Lower compared with higher educational attainment was associated with 4 to 7-fold increased likelihood of belonging to DP1 (p<0.001) and 2 to 3-fold increased likelihood of belonging to DP3 (p = 0.001) among community-dwelling participants (S4 Table).

## Discussion

Utilising dietary data collected by 24-hr MPR on two occasions from participants in the Newcastle 85+ Study, we identified three unique DP in very old adults living in the community including those in institutions in the North East of England. DP varied with key sociodemographic, lifestyle, health and functioning factors. ‘High Red Meat’ (DP1) was characterised by higher intake of red meats/meat dishes (pork, beef, lamb), gravy, potato/potato dishes, unsaturated fats spreads and lower intake of butter, and had the highest percent energy from protein and starch. ‘Low Meat’ (DP2) was under-represented by potentially less healthy foods (e.g. red meat/meat dishes, potato/potato dishes), and over-represented in healthier foods (e.g. fruits, whole grains, fish). Participants in the ‘High Butter’ (DP3) cluster were most likely to be butter consumers, had very low intake of unsaturated fat spreads, and had the highest percent energy from total fat and SFA. Compared with DP1 and DP3, participants in DP2 were healthier (i.e. less cognitive impairment and depressive symptoms) and less disabled, and were more advantaged in all three indicators of SES (i.e. education, occupation, and deprivation index). Membership of this favourable DP was predicted by higher educational attainment irrespective of other SES measures and important covariates, and was unrelated to the type of residence.

Diet and dietary patterns (DP) as modifiable lifestyle factors have been intensively investigated in relation to various health outcomes in middle and later life, but the extent to which diet/DP affects health and functioning in the very old (those aged 85+) is poorly understood. Equally, there is a paucity of data addressing the socioeconomic (SES) determinants of dietary choices in this age group. To our knowledge, the present study is the first cohort study to show associations between education and dietary habits in very late life. This apparent effect of education was in addition to other SES advantages associated with higher social class (occupation) and the prosperity of the area of residency.

Because of the lack of studies evaluating diet in the very old, we contrasted our findings with the results from several recent studies conducted in Europe and North America which included older adults aged 85+ and employed *a posteriori* (exploratory) approaches to derive DP. However, dissimilarities in the methodology for dietary assessment (e.g. food frequency questionnaires (FFQ) versus 24-hr MPR) and the exploratory statistical techniques (e.g. factor analysis versus clustering) used to characterise DP in a specific population of older adults make these comparisons difficult—a recognized disadvantage of *a posteriori* approaches to DP analysis [45]. We opted for a clustering method (i.e. Two-Step) which described total, habitual dietary patterns in this cohort by creating unique and mutually exclusive dietary groups based on similarities of food/nutrient intake of individuals within each DP [29]. As a composite measure for complex combinations of foods/nutrients, such DP can act as a single measure of dietary exposure which can be linked with various health outcomes, lifestyle, and SES determinants. However, the utility of the derived DP depends critically on the robustness of the dietary assessment method used to collect the primary dietary data and the resultant clustering variables [27,28]. To assess diet in the very old we employed the 24-hr MPR, which was validated for use in this age group in a pilot study, and yielded more realistic estimates of energy and nutrients compared with a FFQ [33].

Despite some methodological differences across these studies, and demographic, cultural and geographic dissimilarities of the populations included, ‘healthier’ DP were apparent in all studies which examined associations with health-promoting behaviours and with higher SES (i.e. higher education and income) [10–13,22,46]. On the other hand, ‘less healthy’ DP dominated among older adults with lower educational attainment and less income [10,12,46] (S5 Table).

SES inequality in health and diet quality associated with differences in education, social class (occupation), and income (or combinations of these measures) have been well researched in the general and older adult populations (aged 60+) [10,13,14,18,21–25]. Although each SES indicator is conceptually different and explains a unique portion of SES variability in relation to diet, most studies do not attempt to adjust simultaneously for all SES factors due to either data limitations or lack of a theoretical concept that would justify the use of selected measures [47]. In the present study, we used a lifespan perspective and investigated the combined associations between dietary pattern and three SES indicators that spanned more than 60 years from early adulthood (education), through mid to late (occupation/social class) until very late adulthood (deprivation index). Each indicator covers a different portion of SES and, prior to this study, their cumulative effect on dietary patterns in the very late life has not been investigated. After adjusting for other SES measures and for important covariates, we found that only higher education attainment strongly predicted membership of the healthier DP (DP2) independently of the type of residency (i.e. living in institution or not). This finding is in agreement with the cumulative disadvantage hypothesis which posits that the negative effect of low education attainment on health and health-related behaviors (e.g. diet) increases with age [48]. In relation to health, higher education not only offers possibilities for greater economic resources and higher social status associated with more prestigious occupation, but also may provide greater interest in health risks and in implementing sustained healthier lifestyles which enhance the individual’s sense of control over life outcomes [49,50]. Whilst this emphasises the likely importance of education on lifelong health, it should not be seen as an impediment to the development of suitably-tailored interventions to promote healthier dietary choices in later life [51,52], and consequently to preserve and improve health and functioning of very old ‘survivors’.

This is the first study to characterise the dietary patterns of the very old (aged 85+), including those in institutions. Strengths of this study include: (a) diet assessment by the 24-hr MPR which has been validated in this cohort/age group [33]; (b) use of an exploratory approach and clustering analysis to derive DP, which provided descriptions of the habitual dietary behaviours of the target population; (c) use of several analytic techniques to ensure the robustness of the final DP solution, and (e) use of a range SES indicators across the lifespan.

The present analysis is limited by its cross-sectional design and by a single measure of dietary exposure—therefore no causality can be inferred. As the DP derived here are based on the dietary intake of the population under study, our findings may not be generalizable to other populations of very old adults who have different dietary habits for cultural or other reasons. Although the 24-hr MPR provided a better estimate of energy intake (EI) than the FFQ in this cohort [33], dietary mis-reporting remains a potentially significant issue [53]. The Cardiovascular Seniors and Built Environment Study of older adults aged 60 to 99 [20] excluded those reporting <500 kcal and >5000 kcal per day prior to DP analysis, although in our sample we had no participants below 500 or above 5000 kcal. Use of biomarker-based approaches which do not rely on self-reported dietary intake may enhance the robustness of future dietary assessments in very old people [54]. We used the IMD as a more current SES indicator in very late life. However the IMD is a weighted, aggregated *area* level of deprivation in seven domains [38], and therefore does not conceptualise individual experiences of deprivation. Lastly, our sample as other late-life cohorts, may be underrepresented in older adults with low SES who may have experienced increased mortality by the age of 85, and therefore our sample may have reduced power to detect SES differences.

To summarise, we established three distinct DP in the very old adults living in the NE England, which varied by sociodemographic, health and functioning, lifestyle and nutritional factors. Higher educational attainment was strongly associated with a healthier diet 60+ years later above and beyond advantages provided by higher social class (occupation) and the affluence of residential area. Future studies in this cohort will determine associations between DP and health outcomes over time, in particular whether these DP appear to modulate trajectories in cognition, disability and frailty beyond the age of 85 years.

## Supporting Information

S1 Table Food items included in 30 food group used in the cluster analysis. (DOCX)

S2 Table Mean daily intake (g) of 10 food groups with highest to lowest importance factor by dietary patterns. (DOCX)

S3 Table Major baseline chronic diseases by dietary patterns. (DOCX)

S4 Table Socioeconomic determinants of dietary pattern membership in community-dwellers. (DOCX)

S5 Table Selected studies of older adults reporting inverse relationship between DP derived *a posteriori* and SES indicators. (DOCX)
