# Supplementary material for: Dietary Patterns and Socioeconomic Status in the Very Old: The Newcastle 85+ Study
Source: PLoS One. 2015 Oct 21;10(10):e0139713. doi: 10.1371/journal.pone.0139713 (PMC4619552; doi:10.1371/journal.pone.0139713)
Supplement: S1 Table — (DOCX) [file pone.0139713.s001.docx]

**S1Table.** Food items included in 30 food group used in the cluster analysis*.

| **Food Group** (n=30) | **Included food items** |
| --- | --- |
| Fruits | fresh fruits, dried fruits, 100% fruit juices, canned/stewed fruits (e.g. peaches, prunes |
|  | and plums, pineapple, fruit cocktail) |
| Vegetables | all vegetables (fresh and frozen, green leafy, root, cruciferous, green salads), |
|  | vegetable stews and dishes (includes curry, vegetable stir fry mix, sauerkraut, |
|  | casserole, ratatouille) |
| Potatoes and potato dishes | old and young potatoes (boiled, baked, roasted, mashed, French-fried), sweet |
|  | potatoes, non-meat potato dishes (potato salad, potato pie) |
| Legumes | pulses, lentils, peas (split and dried) and beans (includes baked beans) |
| Nuts | nuts and seeds |
| Refined grains and cereal products | white bread and rolls, rice, pasta, breakfast cereal with sugar, tinned cereal |
| Whole grains and cereal products | brown bread, wholemeal cereal |
| Fish and seafood | oily fish (includes canned tuna and mackerel), white fish, fish in batter/bread |
|  | crumbs, shellfish |
| Red meats and meat dishes | beef and beef dishes (includes minced beef), pork and pork dishes, lamb and lab |
|  | dishes, combined meat dishes (includes meat with pasta, meat pies and pastries) |
| Bacon and ham | bacon and ham (grilled, fried, raw, smoked) |
| Poultry | chicken and turkey in batter, chicken and turkey dishes |
| Processed and other meats | sausages, burgers, kebabs, game, organ meats |
| Eggs | eggs and egg dishes (e.g. flan, quiche) |
| Soups | vegetable- and meat-based soups |
| Butter | butter and butter-based blended spreads |
| Saturated fat spreads and margarines | dairy fat spreads, margarine and vegetable oils fat spreads, lard |
| Unsaturated fat spreads and oils | poly- and monounsaturated fat spreads, oils (olive oil, sunflower oil) |
| Gravy | home-made gravy, gravy from granules |
| Low fat dairy | low fat milk, semi-skimmed milk, low fat cheese, low fat yoghurt |
| High fat dairy | whole milk, milk shakes, cream, high fat cheese, processed cheeses, cheese dishes |
| Preserves and syrups | jam, marmalade, honey, syrups, fruit spread, ice cream topping |
| Chocolate | chocolate, chocolate-covered bars |
| Biscuits and cakes | sweet biscuits (homemade, Digestive, Jaffa), coffee and tea cakes, sweet pastries, |
|  | other cakes (e.g. fruit cake, doughnuts, sponge cake, waffles, scones, jam tarts) |
| Desserts and sweets | ice cream, ice cream desserts and lollies, non-chocolate sweets (fudge, |
|  | marshmallow), milk-based sweet pudding (e.g. custard, rice pudding), nonmilk- |
|  | based puddings (e.g. apple pie, fruit mousse, jelly, flan, pancakes) |
| Snacks and savouries | savoury biscuits, potato crisps, non-potato snacks (e.g. pretzels, corn snacks), cereal- |
|  | based savouries with vegetable or meat addition (e.g. meat spring rolls, falafel, |
|  | samosas) |
| Tea | infusion (black green, herbal), excluded added sugar but with whole or semi- |
|  | skimmed milk |
| Coffee | infusion or instant (excluding added sugar but with whole or semi-skimmed milk) |
| Alcohol | all alcoholic beverages (beer, lager, spirits, vine, liquors) |
| Hot drinks | other hot drinks water- or milk-based (e.g. hot chocolate drink) |
| Soft sugary drinks | carbonated drinks, cordials and squashes |

^*^Human Nutrition Research Centre (HNRC) Newcastle University 118 food groups [36] were further combined into 33 food groups based on food and nutrient composition similarities. Food groups which were not combined included salt, herbs and spices, nutritional supplements, and miscellaneous food items from food recipes [e.g. yeast extract, wheat germ, stock cubes, meat extracts, stuffing, etc.]). Of 33 food groups, three (water, table sugar and salad dressings) were excluded from final dietary pattern (DP) solution because of consistently low and non-significant contribution to DP separation, stability and robustness.
