# Supplementary material for: Dietary Patterns and Socioeconomic Status in the Very Old: The Newcastle 85+ Study
Source: PLoS One. 2015 Oct 21;10(10):e0139713. doi: 10.1371/journal.pone.0139713 (PMC4619552; doi:10.1371/journal.pone.0139713)
Supplement: S2 Table — (DOCX) [file pone.0139713.s002.docx]

**S2Table.** Mean daily intake (g) of 10 food groups with highest to lowest importance factor by dietary patterns.

| **Food Group** | All | DP1: High Red Meat | DP2: Low Meat | DP3: High Butter | p^*^ |
| --- | --- | --- | --- | --- | --- |
|  | n=793 | n=277 | n=260 | n=256 |  |
| Butter | 8.75 (14.35)^†^ | 1.15 (5.64) | 5.80 (11.83) | 19.95 (16.30) | <0.001 |
| Unsaturated fat spreads | 8.58 (13.39) | 12.88 (14.51) | 11.64 (14.80) | 0.82 (4.03) | <0.001 |
| Gravy | 14.50 (27.84) | 24.31 (33.31) | 2.20 (12.02) | 16.38 (28.22) | <0.001 |
| Potato and potato dishes | 95.21 (79.78) | 117.10 (73.79) | 61.60 (79.06) | 105.67 (75.80) | <0.001 |
| Red meats and meat dishes | 73.96 (82.83) | 91.86 (77.36) | 58.47 (92.45) | 70.36 (74.46) | <0.001 |
| Legumes | 17.27 (29.28) | 23.72 (31.45) | 7.73 (24.69) | 19.97 (28.78) | <0.001 |
| Coffee | 152.00 (222.24) | 123.45 (222.17) | 206.35 (204.28) | 127.68 (230.42) | <0.001 |
| Snacks and savouries | 5.49 (11.11) | 2.45 (6.71) | 8.50 (13.53) | 5.73 (11.37) | <0.001 |
| Vegetables | 89.21 (80.83) | 98.15 (79.72) | 76.17 (87.02) | 92.77 (73.81) | <0.001 |
| Low fat dairy | 75.20 (99.99) | 81.70 (108.05) | 88.55 (98.46) | 54.62 (88.99) | <0.001 |

*Kruskal-Wallis test.

^†^Values are means (SD) in grams.
