# Supplementary material for: Dietary Patterns and Socioeconomic Status in the Very Old: The Newcastle 85+ Study
Source: PLoS One. 2015 Oct 21;10(10):e0139713. doi: 10.1371/journal.pone.0139713 (PMC4619552; doi:10.1371/journal.pone.0139713)
Supplement: S3 Table — (DOCX) [file pone.0139713.s003.docx]

**S3Table.** Major baseline chronic disease by dietary patterns.

| **Chronic diseases** | All participants* | DP1: High Red Meat | DP2: Low Meat | DP3: High Butter | p^†^ |
| --- | --- | --- | --- | --- | --- |
| Cardiovascular disease^‡^ % (n) |  |  |  |  | 0.04 |
| Yes | 72.0 (608) | 77.5 (214) | 70.8 (184) | 67.8 (173) |  |
| Cerebrovascular disease % (n) |  |  |  |  |  |
| Yes | 20.1 (159) | 22.1 (61) | 19.6 (51) | 18.4 (47) |  |
| Diabetes % (n) |  |  |  |  |  |
| Yes | 13.8 (109) | 17.0 (47) | 10.8 (28) | 13.3 (34) |  |
| Osteoporosis % (n) |  |  |  |  |  |
| Yes | 12.9 (102) | 14.5 (40) | 7.5 (29) | 11.0 (11) |  |
| Dementia % (n) |  |  |  |  | 0.001 |
| Yes | 7.8 (62) | 12.0 (33) | 3.5 (9) | 7.8 (20) |  |
| *APOE ε4* status |  |  |  |  | 0.002 |
| 1+ *ε4* allele | 26.7 (161) | 32.7 (68) | 17.9 (36) | 29.5 (57) |  |
| Osteoarthritis-related diseases % (n) |  |  |  |  |  |
| Yes | 55.9 (442) | 55.4 (153) | 57.3 (149) | 54.9 (140) |  |
| Inflammatory arthritis % (n) |  |  |  |  |  |
| Yes | 3.9 (31) | 4.3 (12) | 2.7 (7) | 4.7 (12) |  |
| COPD % (n) |  |  |  |  |  |
| Yes | 16.6 (131) | 15.2 (42) | 16.2 (42) | 18.4 (47) |  |
| Asthma % (n) |  |  |  |  |  |
| Yes | 4.2 (33) | 2.9 (8) | 5.8 (15) | 3.9 (10) |  |
| Thyroid disease % (n) |  |  |  |  |  |
| Yes | 14.9 (118) | 12.3 (34) | 14.6 (38) | 18.0 (46) |  |
| Cancer % (n) |  |  |  |  |  |
| Yes | 6.4 (51) | 4.7 (13) | 7.7 (20) | 7.1 (18) |  |
| Renal impairment %(n) |  |  |  |  |  |
| Yes | 22.8 (180) | 26.9 (70) | 17.3 (45) | 27.1 (65) |  |

COPD, chronic obstructive pulmonary disease.

^*^Two participants with assigned dietary pattern did not have complete health assessments and GP records data, and were excluded from the analyses.

^†^Kruskal-Wallis test for ordered and χ^2^ test for categorical variables. In the *post hoc* χ^2^ test analyses, adjusted residuals were used to determine which cells were major contributors to rejecting the null hypothesis at α=0.05.

^‡^Includes hypertension, cardiac disease, and peripheral vascular disease.
