# Supplementary material for: Dietary Patterns and Socioeconomic Status in the Very Old: The Newcastle 85+ Study
Source: PLoS One. 2015 Oct 21;10(10):e0139713. doi: 10.1371/journal.pone.0139713 (PMC4619552; doi:10.1371/journal.pone.0139713)
Supplement: S4 Table — (DOCX) [file pone.0139713.s004.docx]

**S4Table.** Socioeconomic determinants of dietary pattern membership in community-dwellers***.**

| **Dietary Pattern** | SES indicator | Model 1^†^ | p | Model 2^‡^ | p | Model 3^§^ | p |
| --- | --- | --- | --- | --- | --- | --- | --- |
| DP1: High Red Meat | Education (years) | OR (95% CI) |  | OR (95% CI) |  | OR (95% CI) |  |
|  | 0-9 | 5.89 (3.09-11.21) | <0.001 | 6.62 (3.33-13.14) | <0.001 | 6.69 (3.36-13.31) | <0.001 |
|  | 10-11 | 3.05 (1.50-6.21) | 0.002 | 3.84 (1.81-8.14) | <0.001 | 3.83 (1.80-8.16) | <0.001 |
|  | ≥12 (ref) | 1 |  | 1 |  |  |  |
|  | Occupational class |  |  |  |  |  |  |
|  | Routine/manual | 2.28 (1.53-3.41) | <0.001 |  |  |  |  |
|  | Intermediate | 1.54 (0.88-2.69) | 0.13 |  |  |  |  |
|  | Managerial/administrative (ref) | 1 |  |  |  |  |  |
|  | Deprivation Index |  |  |  |  |  |  |
|  | Poor areas | 2.28 (1.36-3.81) | 0.002 |  |  |  |  |
|  | Intermediate | 1.25 (0.82-1.90) | 0.3 |  |  |  |  |
|  | Affluent areas (ref) | 1 |  |  |  |  |  |
| DP2: Low Meat (ref) |  | 1 |  | 1 |  | 1 |  |
| DP3: High Butter | Education (years) |  |  |  |  |  |  |
|  | 0-9 | 3.31 (1.87-5.86) | <0.001 | 3.37 (1.89-6.04) | <0.001 | 3.41 (1.90-6.12) | <0.001 |
|  | 10-11 | 2.77 (1.48-5.18) | 0.001 | 2.96 (1.56-5.62) | 0.001 | 2.92 (1.53-5.56) | 0.001 |
|  | ≥12 (ref) | 1 |  |  |  |  |  |
|  | Occupational class |  |  |  |  |  |  |
|  | Routine/manual | 2.22 (1.48-3.33) | <0.001 |  |  |  |  |
|  | Intermediate | 1.38 (0.76-2.44) | 0.28 |  |  |  |  |
|  | Managerial/administrative (ref) | 1 |  |  |  |  |  |
|  | Deprivation Index |  |  |  |  |  |  |
|  | Poor areas | 2.00 (1.18-3.40) | 0.01 |  |  |  |  |
|  | Intermediate | 1.34 (0.88-2.05) | 0.17 |  |  |  |  |
|  | Affluent areas (ref) | 1 |  |  |  |  |  |

ref (referent)

^*^Multinomial logistic regression with stepwise forward entry. Seventy participants (8.9%) living in institutions were excluded from the analysis.

^†^each SES indicator entered separately

^‡^SES indicators entered together

^§^additionally adjusted for lifestyle (physical activity, smoking) and health-related factors (BMI, cognitive status)
