# Supplementary material for: Dietary Patterns and Socioeconomic Status in the Very Old: The Newcastle 85+ Study
Source: PLoS One. 2015 Oct 21;10(10):e0139713. doi: 10.1371/journal.pone.0139713 (PMC4619552; doi:10.1371/journal.pone.0139713)
Supplement: S5 Table — (DOCX) [file pone.0139713.s005.docx]

**S5Table.** Selected studies of older adults reporting inverse relationship between DP derived *a posteriori* and SES indicators.

| **Study population** | Dietary assessment | DP analyses | Key findings | Reference |
| --- | --- | --- | --- | --- |
| EPIC-Elderly cohort from | FFQ | Principal component and | ‘Sweet and fat-dominated’ DP preferred by | 10 |
| nine European countries, |  | clustering (Ward’s agglomerative | older Europeans (including UK sub-sample) |  |
| aged 60+ (n=99,744) |  | method) | with a **low educational attainment** and |  |
|  |  |  | ‘Vegetable-based’ DP associated with a |  |
|  |  |  | **higher education**; results confirmed with |  |
|  |  |  | clustering analysis |  |
| The University of | three-day 24-hour | latent class clustering analysis | ‘More healthful’ DP (higher intake of fruits, | 12 |
| Alabama at Birmingham | dietary recalls | (i.e. finite mixture modelling) | vegetable, whole grains, nuts, eggs, dairy, |  |
| Study of Aging, aged 65+ |  |  | and legumes) associated with a **higher level** |  |
| (n=622) |  |  | **of education** and **income** compared with |  |
|  |  |  | ‘Western-like’ DP (high intake of starchy |  |
|  |  |  | vegetable, refined grains, meats, fried |  |
|  |  |  | poultry and fish, oils and fats) and ‘Low |  |
|  |  |  | produce, high sweet’ (lowest in fruits and |  |
|  |  |  | vegetables, and highest in sweets) |  |
| The National Diet and | 4-day weighted | hierarchical agglomerative method | ‘Healthy’ DP (lower intake of red meats, | 46 |
| Nutritional Survey UK, | dietary records | (separate DP for men and women) | bacon and ham [both men and women], and |  |
| aged 65+ (n=1097) |  |  | higher consumption of alcohol [men]) |  |
|  |  |  | associated with higher **social class**, **income**, |  |
|  |  |  | and **education**; ‘Traditional’ DPs (high |  |
|  |  |  | intake of cakes and pastries, moderately- |  |
|  |  |  | high in meat, potato, fruits and vegetable |  |
|  |  |  | [both men and women] associated with |  |
|  |  |  | **lower social class**) |  |

SES indicators, socio-economic indicators (education, social class, income); DP, dietary patterns; EPIC, European Prospective Investigation into Cancer and Nutrition; FFQ, food frequency questionnaire.
